# Deficiency of ROS-Activated TRPM2 Channel Protects Neurons from Cerebral Ischemia-Reperfusion Injury through Upregulating Autophagy

**DOI:** 10.1155/2021/7356266

**Published:** 2021-07-27

**Authors:** Xupang Hu, Lijuan Wu, Xingyu Liu, Yi Zhang, Min Xu, Qiuyuan Fang, Lin Lu, Jianguo Niu, Tarek Mohamed Abd El-Aziz, Lin-Hua Jiang, Fangfang Li, Wei Yang

**Affiliations:** ^1^Department of Biophysics, and Department of Neurosurgery of the First Affiliated Hospital, Zhejiang University School of Medicine, Hangzhou 310058, China; ^2^School of Medicine, Taizhou University, Taizhou 318000, China; ^3^School of Medicine, Zhejiang University City College, Hangzhou 310015, China; ^4^Ningxia Key Laboratory of Craniocerebral Diseases, Ningxia Medical University, Yinchuan 750004, China; ^5^Department of Cellular and Integrative Physiology, University of Texas Health Science Center at San Antonio, San Antonio, TX 78229-3900, USA; ^6^Zoology Department, Faculty of Science, Minia University, El-Minia 61519, Egypt; ^7^School of Biomedical Sciences, Faculty of Biological Sciences, University of Leeds, Leeds, UK

## Abstract

Cerebral ischemia-reperfusion (I-R) transiently increased autophagy by producing excessively reactive oxygen species (ROS); on the other hand, activated autophagy would remove ROS-damaged mitochondria and proteins, which led to cell survival. However, the regulation mechanism of autophagy activity during cerebral I-R is still unclear. In this study, we found that deficiency of the TRPM2 channel which is a ROS sensor significantly decreased I-R-induced neuronal damage. I-R transiently increased autophagy activity both *in vitro* and *in vivo*. More importantly, TRPM2 deficiency decreased I-R-induced neurological deficit score and infarct volume. Interestingly, our results indicated that TRPM2 deficiency could further activate AMPK rather than Beclin1 activity, suggesting that TRPM2 inhibits autophagy by regulating the AMPK/mTOR pathway in I-R. In conclusion, our study reveals that ROS-activated TRPM2 inhibits autophagy by downregulating the AMPK/mTOR pathway, which results in neuronal death induced by cerebral I-R, further supporting that TRPM2 might be a potential drug target for cerebral ischemic injury therapy.

## 1. Introduction

The balance of reactive oxygen species (ROS) production and elimination maintains the cellular redox homeostasis, which is vital to cell survival in various physiological and pathological conditions [1]. Cerebral ischemia-reperfusion (I-R) is a clinical disorder characterized by severe neurological dysfunctions, which is a leading cause of long-term disability and death globally [2]. Due to the drastic interruption of blood supply, followed by reperfusion, the balance of generation of excessive ROS and the tissue's ability to detoxify ROS are disrupted. The I-R injury was found dependent on the reintroduction of oxygen, indicating that ROS would possibly be the main cause of neuronal death in reperfusion injury [3]. ROS overproduction leading to cell death was a complex mode involving necrosis, autophagy, and apoptosis in I-R injury [4].

As a ROS sensor, the TRPM2 channel is a Ca^2+^ permeable nonselective cation channel and is activated by the adenosine diphosphate-ribose (ADPR) [5] that is generated through poly(ADPR)polymerase (PARP)/poly(ADPR)glycohydrolase (PARG) pathway [6]. Recent studies have linked TRPM2 to cerebral I-R injury. For example, TRPM2 deficiency or pharmacological inhibition of TRPM2 prevented oxygen and glucose deprivation reperfusion- (OGD-R-) induced neuronal death in cultured mouse cortical neurons [7, 8]. In addition, the neuronal death of TRPM2 knockout (TRPM2 KO) mice induced by the transient middle cerebral artery occlusion (tMCAO) model was substantially attenuated compared to that of WT mice [9]. In addition, previous studies reported that TRPM2 mediated neuronal death in I-R by resulting in Zn^2+^ accumulation [10], regulating NMDAR expression level [11] and inducing inflammation [12]. Since brain I-R injury is a complex pathological process, it is still important to explore whether TRPM2 mediated I-R injury by regulating other critical pathways.

Accumulating evidence has indicated that autophagy plays a critical role during cerebral I-R injury. For example, inhibition of autophagy in the reperfusion phase reinforces I-R injury [13], indicating a protective role that autophagy plays in cerebral I-R. In contrast, a recent study reported that Beclin1 deficiency or RNAi knockdown protected the neuronal death in the tMCAO model, suggesting that Beclin1-dependent autophagy mediated the neuronal damage during I-R injury [14]. In addition to Beclin1, it has been demonstrated that hypoxia or ischemia enhances AMP-activated protein kinase (AMPK) activity [15], which further inhibited its downstream protein mammalian target of rapamycin (mTOR) to enhance autophagy [16]. Therefore, modulating autophagy and its upregulated molecular pathways may be beneficial for the treatment of ischemic stroke. Interestingly, recent studies reported that TRPM2 regulates autophagy [17, 18]. For example, a recent study found that TRPM2 deficiency inhibited H_2_O_2_-induced AMPK phosphorylation in neutrophils, suggesting the regulation of the AMPK signaling pathway by TRPM2 [17]. Besides, a recent study reported that TRPM2 channel activation mediated calcium influx, which activated calcium/calmodulin-dependent protein kinase II (CaMK II) and subsequently phosphorylated Beclin1 to inhibit autophagy [18]. It will be attractive to determine whether TRPM2 mediates I-R injury by regulating autophagy.

In this study, we investigated the role of TRPM2 in autophagy-dependent cerebral I-R. The underlying rationale was that I-R-induced oxidative stress activates TRPM2 channels [19], and TRPM2 activation leads to Ca^2+^ influx, which in turn affects autophagy flux [18]. Our results demonstrated that the ROS-activated TRPM2 channel impairs autophagy induction by regulating the AMPK/mTOR pathway during cerebral I-R.

## 2. Materials and Methods

### 2.1. Animals

The TRPM2^−/−^ mice were generated in University of Leeds [20] and the mice used in this study were bred in Zhejiang University. All experiments were approved by and conducted following the ethical guidelines of the Zhejiang University Animal Experimentation Committee.

### 2.2. Antibodies and Reagents

Antibodies against Cathepsin B (1 : 1000, 21718S), cleaved caspase 3 (1 : 1000, Asp175, #9661), caspase 3 (1 : 1000, #9662), phosphorylated-AMPK, AMPK (1 : 1000, 2532S), phosphorylated-mTOR (1 : 1000, #2974), and mTOR (1 : 1000, #2983) were purchased from Cell Signaling Technology (CST, US). Secondary antibodies (1 : 2000) conjugated to horseradish peroxidase were purchased from Santa Cruz Biotechnology. All chemicals were obtained from Sigma-Aldrich or MedChemExpress unless stated otherwise. Stock solutions of compounds were prepared in dimethylsulphoxide (DMSO) or water. The stock chemicals were stored as aliquots at -20°C.

### 2.3. Preparation of tMCAO Mouse Model

Microsurgery of tMCAO was conducted on the 8-week-old wild-type (C57BL/6) or TRPM2 knockout male mice. Mice were anesthetized for surgery via inhalation of isoflurane. We utilized laser Doppler flowmetry (Moor Instruments, Devon, UK) to monitor the cerebral blood flow (CBF) in the territory of the middle cerebral artery (MCA). Animals with less than 80% reduction in CBF in the core of the MCA territory were excluded from the study.

Transient focal cerebral ischemia was induced by MCAO as described previously [21–23]. Briefly, a 6–0 nylon monofilament suture was inserted into the internal carotid to occlude the origin of the MCA. The nylon suture was removed after 1.5 hours to allow reperfusion. Mice were given a tail vein injection of LY294002 (MCE, HY-10108, 5 mg/kg) or intracerebroventricular injection of 7.5 *μ*g 3-methyladenine (3-MA, Sigma, M9281) at the beginning of reperfusion. The same volume of PBS was given to control mice. After the completion of microsurgery, mice were transferred to a heated cage (37°C) for recovery and then transferred to their cages.

### 2.4. Evaluation of Neurological Deficit Score and Infarct Volume

Neurological deficit scores were evaluated after 24 h of reperfusion according to the following rule: 0, no deficit; 1, failure of extending of contralateral forelimb; 2, circling to the right side; 3, falling to the contralateral side; and 4, lacking spontaneous motor activity. After evaluation of the neurological deficit score, the mice were sacrificed, and brain tissue was dissected. After storing at -20°C for 30 minutes, infarct volumes were stained with 2,3,5-triphenyltetrazolium hydrochloride (TTC; 0.25%; Sigma, T8877) and fixed in 4% paraformaldehyde overnight. Finally, the infarct areas were measured with the ImageJ software (National Institutes of Health) and determined by a researcher blinded to the experiments.

### 2.5. Viral Injection

Five-week-old mice were prepared for stereotaxic injection. The detailed protocols have been described previously [24] with minor modifications. Briefly, animals were placed into the anesthesia induction chamber with 2.5% isoflurane and then immobilized on a stereotaxic apparatus. A volume of GFP-mRFP-LC3-AAV virus solution was injected into the bilateral cortex region (1 *μ*l, bregma = 0.20 mm; lateral ± 3.20 mm; ventral 1.50 mm) using a microinjector with a glass electrode at a rate of 0.1 *μ*l min^−1^ with an infusion pump. The syringe needle was left in place for 10 min and withdrawn, and the injection repeated in the opposite hemisphere and then suturing the skin of the head by applying iodophor for disinfection. The mice were placed in a clean cage and with access to water and food ad lib for 3 weeks until the virus expressed for tMCAO. Three weeks later, the tMCAO was performed on these mice. Animals that had undergone tMCAO microsurgery were deeply anesthetized with pentobarbital (100 mg kg^–1^, i.p.) and perfused transcranial with saline followed by 4% PFA in 0.1 M PBS, pH 7.4. Brains were removed, postfixed overnight in 4% PFA at 4°C and transferred to 30% sucrose in 0.1 M PBS, pH 7.4. Coronal sections (50 *μ*m) were cut on a cryostat (Leica CM3050 S, Leica Biosystems, IL, USA) and stored in 0.1 M PBS. Sections were then mounted on DAPI-Fluoromount-G overnight. The images were taken using a LSM880 inverted confocal microscope fitted with a ×63 oil objective (LSM880, Carl Zeiss, Jena, Germany) and appropriate excitation (GFP, 494 nm; mRFP, 548 nm) and emission (GFP, 519 nm; mRFP, 562 nm) wavelengths.

### 2.6. Primary Cortical Neuronal Cell Culture and OGD Reperfusion Procedures

The dissected cortex from embryonic day 17 fetal mice were digested with 0.25% trypsin (Invitrogen; 25200-056) for 8 minutes. Then, Dulbecco's modified Eagle's medium (DMEM) supplemented with 20% fetal bovine serum (FBS) was added to inactivate trypsin. The cell suspension was obtained with triturating 2-3 times with the above medium. Approximately 10^5^ cells/cm^2^ were seeded onto poly-D-lysine (Sigma; P7405)-coated glass-bottom dishes (Cellvis; D29-20-1-N) for live cell imaging. For Western blot, cells were seeded and cultured in cell culture dishes (Corning). Neurons were grown in Neurobasal medium (Gibco; 21103049) supplemented with 2% B27 Supplement (Gibco; 17504044) and 0.25% GlutaMAX Supplement (Gibco; 35050-061). Cultures were maintained for 8–11 days before treatment.

For OGD treatment, DIV8–11 primary cultured neurons were refreshed with glucose-free DMEM (Gibco; 11966025). Cells were then immediately placed in Hypoxia Incubator Chamber (Stemcell; 27310) loaded with mixed gas containing 5% CO_2_ and 95% N_2_ as indicated. For reperfusion, neurons were refreshed with the normal culture medium.

### 2.7. SH-SY5Y Cell Culture and OGD Reperfusion Procedures

The SH-SY5Y cell lines were seeded onto poly-D-lysine (Sigma; P7405)-coated glass-bottom dishes (Cellvis; D29-20-1-N) for live cell imaging. Cells were grown in DMEM/F12 (Gibco; 11320033) supplemented with 10% FBS (PAN; ST30-3302).

For OGD treatment, cultured cells were refreshed with glucose-free DMEM (Gibco; 11966025). Cells were then immediately placed in Hypoxia Incubator Chamber (Stemcell; 27310) loaded with mixed gas containing 5% CO_2_ and 95% N_2_ for 6 hours. Cells were refreshed with normal culture medium during the reperfusion stage for 6 hours. For live cell autophagy flux experiments, cells were simultaneously incubated with N-(p-amylcinnamoyl) anthranilic acid (ACA, MCE, HY-118628), Torin 1 (MCE, HY-13003), or N-acetyl cysteine (NAC, Sigma, A7250) at its working concentration as indicated during reperfusion stage.

### 2.8. Transient Transfection

Primary cortical neurons were transfected with Mouse Neuron Nucleofector Kit (VPG-1001, Lonza, Basel, Switzerland) according to the manufacturer's protocol. Briefly, 1 × 10^6^ cells were collected during primary neuronal culture and then resuspended with 100 *μ*L Nucleofector Solution containing 4 *μ*g DNA. The cell suspension was transferred into the cuvette and electroporated with Nucleofector Program O-005. Approximately 50% of neurons can be transfected.

### 2.9. Cytosolic ROS Measurements in Primary Cortical Neurons

Cytosolic ROS production was measured by using cell-permeant 2′,7′-dichlorodihydrofluorescein diacetate (H2DCF-DA) (Biotium). Upon cleavage of the acetate groups by intracellular esterase and oxidation, the nonfluorescent H2DCF-DA is converted to the highly fluorescent DCF. Briefly, cells were treated with various agents for the desired length of time and then incubated with HBSS (GIBCO®) containing 10 *μ*M H2DCF-DA for 30 minutes at 37°C/5% CO_2_. Cells (>5000) were collected using ACCUTASE™ (STEMCELL™), washed, and resuspended in PBS prior to analysis using a ACEA NovoCyte™ flow cytometer (Agilent Technologies, No. 205, Zhaohui Road, Hangzhou, Zhejiang, China). Cytometer settings were optimized for green (FITC-H) fluorescence, and data were analyzed with the NovoExpress 1.4.1 Software (Agilent Technologies, No. 205, Zhaohui Road, Hangzhou, Zhejiang, China).

### 2.10. Electron Microscopy

The primary cortical neurons subjected with OGD reperfusion were fixed by 2.5% glutaraldehyde in 0.1 M cacodylate buffer. After rinsing in 0.1 M phosphate buffer (pH 7.3), the samples were postfixed in ferrocyanide-reduced osmium tetroxide. These sections were examined in a FEI Tecnai Spirit (T12) transmission electron microscope with a Gatan US4000 4k × 4k CCD at the Zhejiang University EM core facility.

### 2.11. Live-Cell Imaging of Autophagy Influx

Following the desired treatments, cells were imaged at 37°C using a LSM880 inverted confocal microscope fitted with a ×63 oil objective at appropriate excitation (GFP, 488 nm; mCherry, 548 nm) and emission (GFP, 519 nm; mCherry, 562 nm) wavelengths.

### 2.12. Cathepsin B Activity Assay

Cathepsin B activity was examined by Cathepsin B Assay Kit (Magic Red) (ab270772, Abcam) according to the manufacturer's protocol. Briefly, following the desired treatments cells were loaded with substrate and incubated for 60 min at 37°C protected from light.

### 2.13. Western Blot

Proteins in the lysates of the cortex were separated on 10% SDS-PAGE and subjected to Western blotting using corresponding primary antibodies and HRP-conjugated secondary antibodies. Bands were detected by chemiluminescence and quantified using Image J.

### 2.14. Data Analysis

Colocalization between mRFP-LC3 and GFP-LC3 was analyzed using the Imaris software (Bitplane). All experiments were performed at least three times (*n*), and the values are presented as mean ± SEM or SD. Statistical significance was determined using the Student *t*-test or one-way ANOVA, followed by Tukey's post hoc test. Probability (*p*) values are indicated with ^∗^, ^∗∗^, ^∗∗∗^, and ^∗∗∗∗^, which correspond to values of 0.05, 0.01, 0.001, and 0.0001, respectively.

## 3. Results

### 3.1. ROS Level Has Been Accumulated in I-R Injury, while TRPM2 Deficiency Protected Neurons from I-R Injury

**Figure 1:**
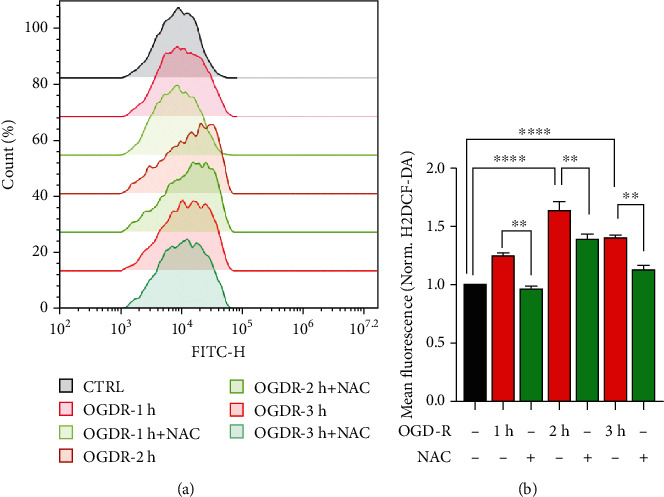
Time-lapse of ROS production under OGD-R treatment. (a) Primary cortical neurons treated with medium alone (CTRL) or 1 h of OGD plus different time (1 h, 2 h, or 3 h) of reperfusion (OGD-R) minus or plus NAC (5 mM) were stained with H2DCF-DA and subjected to FACS (number of cells ≥ 2000). (b) Mean ± SEM data from 2 independent experiments. Each experiment performed 2-3 biological replicates. ^∗∗^*p* < 0.01; ^∗∗∗∗^*p* < 0.0001; one-way ANOVA with post hoc Tukey test.

**Figure 2:**
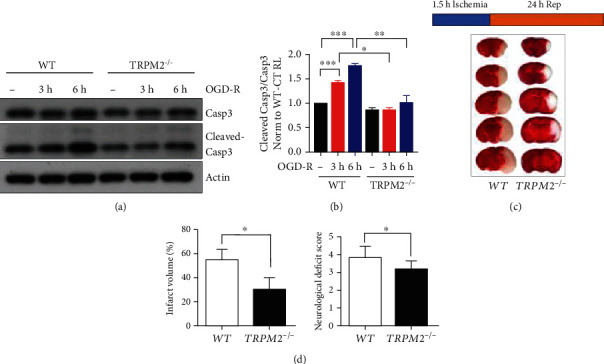
I-R induced neuronal death, which could be rescued by TRPM2 deficiency. (a) Primary cultured cortical neurons were subjected to 1 h of OGD followed by 3 or 6 h of reperfusion (OGD-R 3 h and OGD-R 6 h) at DIV9. The cleaved Casp3, Casp3, and actin levels in cultured neurons were determined by Western blot. (b) Semiquantitative analysis showed the ratio of the cleaved Casp3 and Casp3 band intensities. (c) Mice were subjected to middle cerebral artery occlusion for 1.5 h, and reperfusion was allowed by removing the monofilament suture. Animals were euthanized 24 h after tMCAO, and representative TTC-stained brain slices from each group are shown. (d) Infarct volumes were determined by TTC staining in the bar charts (mean ± SD, *n* = 6). The neurological deficit scores of each group are presented. Data was quantified from 3–4 independent experiments. ^∗^*p* < 0.05; ^∗∗^*p* < 0.01; ^∗∗∗^*p* < 0.001; ne-way ANOVA with post hoc Tukey test.

ROS overproduction has been proposed as the main cause of reperfusion injury, which could further lead to necrosis, autophagy, apoptosis, and necroptosis resulting in organ remodeling [3]. Therefore, firstly, we examined the ROS production during OGD-R treatment. We found that the ROS level had a modest increase since 1 h of reperfusion which was reduced by ROS scavenger N-acetyl-L-cysteine (NAC). Interestingly, the ROS production was significantly induced at 2 h of reperfusion while followed by a slight decrease at 3 h of reperfusion (Figure 1). Besides, we found that the caspase 3 activation was significantly enhanced with OGD-R time at 3 hours and 6 hours (Figures 2(a) and 2(b)). These results indicated that significant neuronal apoptosis and ROS overproduction were caused by I-R injury. Previous studies reported that TRPM2-KO mice protected neurons from I-R injury [10]. To verify this, we also performed the tMCAO experiment and confirmed that TRPM2 deficiency significantly reduced I-R-induced infarct volume and neurological deficit score (Figures 2(c) and 2(d)). Since the mechanism by which TRPM2 deficiency protects neurons from I-R injury remains unclear, we further explored the mechanism by which ROS-activated TRPM2 ion channel mediated neuronal death in I-R.

### 3.2. TRPM2 Deficiency Protected Neurons from I-R Injury in a Promoting Autophagy Induction Manner *In Vitro* and *In Vivo*

**Figure 3:**
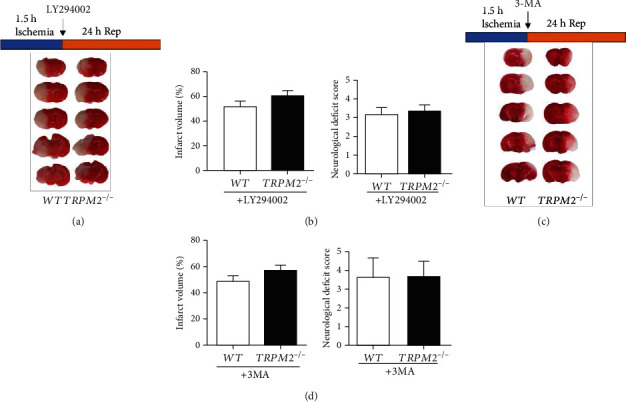
Inhibition of autophagy abolishes the protection of TRPM2 deficiency in I-R injury. Mice were subjected to middle cerebral artery occlusion for 1.5 h, and reperfusion was allowed for 24 h with (a) LY294002 (5 mg/kg) or (c) 3-MA (7.5 *μ*g) by removing the monofilament suture. Animals were euthanized 24 h after tMCAO, and representative TTC-stained brain slices from each group are shown. Infarct volume and neurological deficit score were presented in the bar charts with treatment of (b) LY294002 or (d) 3MA (mean ± SD, *n* = 6). Unpaired Student's *t*-test.

Previous studies have shown that autophagy activity plays a critical role in I-R injury, but which mechanism is still debatable [13]. Since the TRPM2 channel is activated by ROS and ADPR [25], and several studies have suggested TRPM2 can regulate autophagy [26, 27], we first tested whether TRPM2 was involved in autophagy-dependent I-R injury. As expected, TRPM2 deficiency mice strongly reduced the infarct volume and improved neurological deficit score in the tMCAO model, which can be abolished by two autophagy inhibitors, LY294002 and 3-MA (Figure 3). Inhibition of autophagy in the tMCAO wild-type mice *per se* seemed not to alter the infarct volume and neurological deficit score. These data indicated that TRPM2 deficiency protected neurons from I-R injury by increased autophagy activity.

**Figure 4:**
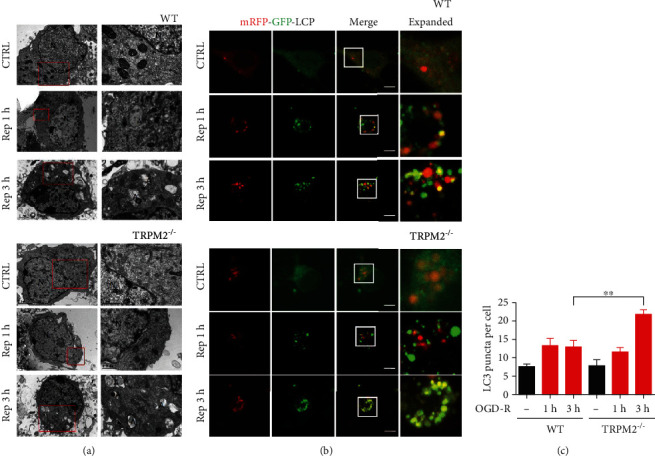
TRPM2 deficiency promotes autophagy induction under OGD-R in primary cortical neurons. (a) Primary cultured neurons from WT and TRPM2 KO mice were subjected to 2 h of OGD plus 1 or 3 h of reperfusion in DIV9. Autophagosome was observed by electron microscopy. Autophagosome was labelled as A and nucleus as N. (b) Primary cultured neurons transfected with mRFP-GFP-LC3 were subjected to 1.5 h of OGD plus 1 or 3 h of reperfusion (OGD-R 1 h and OGD-R 3 h) in DIV9. Representative z-stack confocal images showing the colocalization of mRFP-LC3 with GFP-LC3 induced by OGD-R. (c) Columns represent number of mRFP-GFP-LC3 puncta per cell (mean ± SEM). Scale bars: 10 *μ*m. Data was quantified from 3–4 independent experiments. ^∗∗^*p* < 0.01; one-way ANOVA with post hoc Tukey test.

To further explore how TRPM2 deficiency rescued neuron death through upregulating autophagy, autophagosome was visualized by electronic microscopy. The autophagosome formation was slightly induced at 1 hour of reperfusion both in WT and TRPM2-KO neurons (Figure 4(a)). However, after 3 hours of reperfusion, the number of autophagosome was largely increased. Besides, compared to WT neurons, there were more autophagosomes in TRPM2-KO neurons (Figure 4(a)). These data indicated that TRPM2 deficiency enhanced autophagy induction under OGD-R conditions. To further confirm our findings, we exogenously expressed the tandem mRFP-GFP-LC3 to monitor autophagic flux [28] in cultured primary neurons. GFP fluorescence is quenched upon fusion between autophagosome and lysosome due to the low lysosomal pH, whereas the mRFP signal remains stable at low pH. Therefore, the yellow signal (merged of mRFP and GFP) indicates the presence of autophagosome, while the red signal indicates autolysosome. Our results showed that OGD-R increased the autophagosome formation (GFP+/mRFP+) both in WT and TRPM2-KO neurons in a reperfusion-time-dependent manner (Figure 4(b)). At reperfusion of 1 hour, there is no significant difference between WT and TRPM2-KO neurons. However, at 3 hours of reperfusion, there is more autophagosome (GFP+/mCherry+) in TRPM2-KO neurons compared to WT neurons (Figures 4(b) and 4(c)), suggesting that TRPM2 deficiency upregulated autophagy induction during I-R.

**Figure 5:**
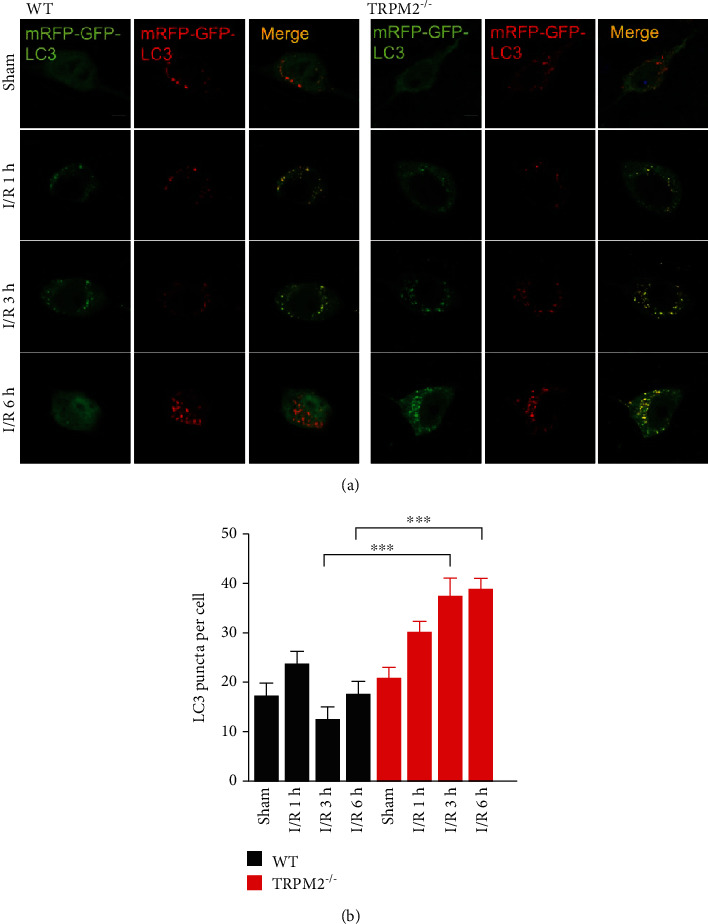
TRPM2 deficiency promotes autophagy induction during I-R injury *in vivo*. (a) WT and TRPM2-KO mice were subjected to sham or MCAO for 1.5 h followed by 1, 3, or 6 h of reperfusion. Then, brain slices were mounted, and confocal images were taken. Representative images of cells expressing AAV-mRFP-GFP-LC3 of brain slices from WT and TRPM2-KO mice are shown. (b) Summary graph showing the quantification of the number for overlapped puncta, as determined by colocalization of all cells from the experiments shown in (a). Columns represent number of mRFP-GFP-LC3 puncta per cell (mean ± SEM). Scale bars: 5 *μ*m. Data was quantified from 3–4 independent experiments. ^∗∗∗^*p* < 0.001; one-way ANOVA with post hoc Tukey test.

To further observe the dynamic autophagic flux of I-R injury *in vivo*, we injected adeno-associated virus (AAV) expressing mRFP-GFP-LC3 into the cortex of WT and TRPM2-KO mice. Fluorescent signals of both mRFP and GFP were detected in the cortical neurons from the sham and I-R groups (Figure 5(a)). Consistent with the results *in vitro*, the number of autophagosome (GFP+/mRFP+) in brain slices was similar between WT and TRPM2-KO mice (Figure 5(b)) at 1 hour of reperfusion, while at 3 hours and 6 hours of reperfusion, the autophagosome number from TRPM-KO mice was largely increased compared to that from WT mice (Figure 5(b)). These data suggested that TRPM2 deficiency reduced I-R injury through autophagy induction *in vitro* and *in vivo*.

### 3.3. TRPM2 Deficiency-Promoted Autophagy Is Mediated by the AMPK/mTOR Pathway to Rescue I-R Injury

A previous study reported that TRPM2 inhibited autophagy through Ca^2+^-CAMK2 cascade-phosphorylated Beclin1 in Hela cells [16]. We examined the phosphorylation level of the Beclin1 Ser295 site and found that this site has no significant difference between ischemic WT and TRPM2-KO neurons (Figure S1), suggested that TRPM2 deficiency promoted I-R-induced autophagy is Beclin1-independent.

**Figure 6:**
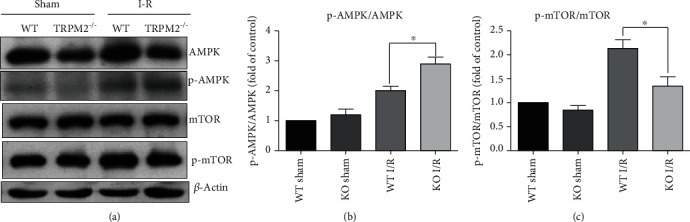
TRPM2 deficiency-promoted autophagy is mediated by the AMPK/mTOR pathway in I-R injury. (a) WT and TRPM2-KO mice were subjected to sham or MCAO for 1.5 h, and reperfusion was allowed for 6 h by removing the monofilament suture. Then, the mice were euthanized, and ischemic penumbra was collected for immunoblot. 3 *μ*g of protein was sequentially immunoblotted with antibodies against AMPK, phosphorylated-AMPK (p-AMPK), mTOR and phosphorylated-mTOR (p-mTOR), and *β*-actin. Semiquantitative analysis of (b) p-AMPK/AMPK and (c) p-mTOR/mTOR is shown. Data was quantified as mean ± SEM from 3-4 independent experiments. ^∗^*p* < 0.05; one-way ANOVA with post hoc Tukey test.

In addition, AMPK has been demonstrated to play a crucial role in autophagy induction in mammalian cells. AMPK could promote autophagy by phosphorylating autophagy-related proteins, such as mTOR, which induced initial phagophore formation [29]. A previous study has reported that the AMPK/mTOR pathway mediates OGD-R-induced cell injury by promoting autophagosome formation [30]. Since our above data has demonstrated that TRPM2 plays an inhibitory role in OGD-R-induced autophagy induction, we are wondering whether TRPM2 affects autophagy by regulating the AMPK/mTOR pathway. Our biochemical data revealed that the level of phosphorylated AMPK was significantly enhanced by the tMCAO treatment in WT mice (Figures 6(a) and 6(b)). Moreover, the phosphorylated AMPK was further upregulated in TRPM-KO mice compared to WT mice (Figures 6(a) and 6(b)), which indicated that TRPM2 activated by I-R injury inhibited the AMPK activation. In contrast, the level of phosphorylated mTOR was reduced in TRPM2-KO mice compared to that in WT mice after the tMCAO treatment (Figures 6(a) and 6(c)), supporting the activation of AMPK activity in TRPM2 deficiency mice after I-R injury.

**Figure 7:**
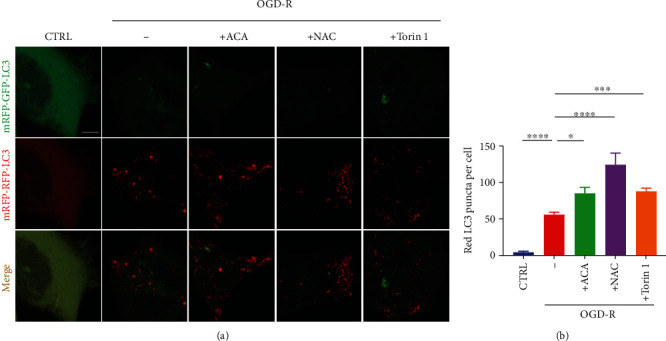
Inhibition of TRPM2 or mTOR enhanced autophagy induction in I-R injury. (a) SH-SY5Y cells overexpressing mRFP-GFP-LC3 were treated with OGD for 6 h and then reperfusion for 6 h with normal medium. 30 *μ* M ACA, 5mM NAC, or 1 *μ*M Torin1 was added during reperfusion. Scale bar: 5 *μ*m. (b) The average numbers of red LC3 puncta per cell in each condition were quantified. All data are represented as mean ± SEM from three independent experiments. ^∗^*p* < 0.05, ^∗∗∗^*p* < 0.001, and ^∗∗∗∗^*p* < 0.0001; one-way ANOVA with post hoc Tukey test.

Finally, to further confirm ROS/TRPM2/mTOR pathway negatively contributed to the autophagy activity in I-R injury, we tracked the autophagy flux by transfecting mRFP-GFP-LC3 into SH-SY5Y cell. Diffused distribution of LC3 at a basal level, but OGD-R treatment increased autolysosome formation (Figure 7(a)). Interestingly, in the presence of ACA or A10 (Figure S2), two TRPM2 inhibitors, the autolysosomes were further elevated under OGD-R (Figure 7). Consistently, NAC, a synthetic precursor of glutathione (GSH) to eliminate ROS, dramatically raised the autophagy flux (Figure 7). Moreover, Torin 1, an mTOR inhibitor, also increased the autolysosome level under OGD-R condition (Figure 7). Taken together, the AMPK/mTOR pathway is responsible for TRPM2-mediated autophagy induction in I-R injury.

## 4. Discussion

In this study, we identified a novel signaling pathway by which TRPM2 mediated I-R injury. Our results demonstrated that OGD-R induced ROS accumulation concomitantly neuronal death, while TRPM2 deficiency attenuated OGD-R-induced neuronal death and I-R injury. Furthermore, TRPM2 deficiency reduced infarct volume and increased neurological deficit score in the tMCAO model, confirming that TRPM2 mediated I-R injury. Interestingly, inhibition of autophagy by 3-MA or LY-294002 abolished the protective effect of TRPM2 deficiency in I-R injury. Further investigation demonstrated that TRPM2 deficiency promoted autophagy induction both *in vitro* and *in vivo*, and this process was mediated by the AMPK/mTOR pathway rather than Beclin1. Our results firstly identified that TRPM2 might impair autophagy to mediate I-R injury. This study pointed towards new therapeutic opportunities for the treatment of cerebral ischemia.

Previous studies have reported that autophagy plays a protective role in I-R injury. Promoting autophagy by urolithin A attenuated renal I-R injury through TFEB-CLEAR (Coordinated Lysosomal Expression and Regulation) pathway [31]. Autophagy protected the heart from I-R injury by attenuating reactive ROS levels and ameliorating mitochondrial dysfunction [32]. Our study revealed that autophagy plays a protective role in TRPM2 deficiency-mediated cerebral I-R injury. Autophagy is a dynamic and multistep process that includes phagophore initiation, autophagosome formation, and fusion with lysosomes for degradation [33]. Therefore, autophagy can be modulated at these steps. In the present study, we found that genetic knockout or chemically inhibition of TRPM2 further upregulates autophagosome formation under I-R *in vivo* or OGD-R treatment *in vitro*. We initially noted that a previous report demonstrated that TRPM2 activation inhibited autophagosome initiation by upregulating phosphorylation of Beclin1 (S295) in Hela cells [18]. Besides, a previous study found that Beclin1 RNAi has a protective role in cerebral I-R [14]. Unfortunately, our result showed there is no difference in phosphorylation of Beclin1 (S295) between WT and TRPM2 KO mice, indicating that TRPM2 regulated autophagy is independent of Beclin1 in cerebral I-R.

AMPK, a critical energy sensor for energy metabolism, has been demonstrated to play an important role in autophagy [34, 35]. Under OGD-R treatment or ischemia, the activation of AMPK in cortical neurons has been reported to exert neuroprotective effects [36]. To get mechanistic insight into whether AMPK is involved in TRPM2-mediated autophagy function, we examined the expression of phosphorylated AMPK levels. Consistent with the previous report, I-R increased phosphorylated AMPK level, and phosphorylated AMPK level of TRPM2 deficiency further increased compared to that of WT mice in cerebral I-R. It is well known that mTOR can be negatively regulated by AMPK in autophagy [37]. Accordingly, our results revealed that TRPM2 deficiency dramatically decreased the phosphorylation level of mTOR protein, which is consistent with the previous study [38]. In addition, our study discovered that inhibiting mTOR also elevated autophagosome formation during OGD-R treatment, further supporting the essential role of mTOR in autophagy. Therefore, we for the first time demonstrated that TRPM2 activation under I-R *in vivo* or OGD-R treatment *in vitro* impaired autophagy by downregulating the AMPK/mTOR pathway. However, the molecular mechanism of the TRPM2 channel regulating the AMPK/mTOR pathway during I-R is still unclear. Actually, in the I-R stage, TRPM2-mediated cellular Ca^2+^ and Zn^2+^ increase have been reported; besides, Ca^2+^ has been demonstrated to regulate the AMPK/mTOR pathway [39, 40]. It will be interesting to uncover the relationship between TRPM2 and AMPK/mTOR signaling pathway under I-R in the future.

## 5. Conclusions

In summary, we confirmed that TRPM2 deficiency reduced ROS-dependent I-R injury and OGD-R-induced neuronal death. We further uncovered that cerebral I-R injury produced ROS-activated TRPM2 channel, which inhibited autophagy induction by attenuating the AMPK/mTOR pathway rather than Beclin1. Although autophagy is a friend or foe during cerebral I-R is still debatable, our results indicated that TRPM2/AMPK/mTOR signaling pathway plays a negative role in autophagy-dependent cerebral I-R injury. Given that upregulation of autophagy is considered a useful strategy to prevent cerebral I-R injury, our findings provide new therapeutic opportunities for ischemic stroke.

## Data Availability

The data used to support the findings of this study are available from the corresponding authors upon request.

## Supplementary Materials

Supplementary Materials Figure S1:TRPM2 deficiency-upregulated autophagy is Beclin1-independent. (a) Primary cultured neurons subjected to medium alone (CTRL), 1 hour of OGD plus 3 hours of reperfusion (OGD-R) at DIV9. 3 *μ*g of protein was sequentially immunoblotted with antibodies against Beclin1, phosphorylated-S295-Beclin1, and *β*-actin. (b) Normalized ratio of the phosphorylated-S295-Beclin1 and Beclin1 band intensities, determined from 3 independent experiments performed as in (a). ^∗∗∗∗^*p* < 0.0001; NS: not significant; one-way ANOVA with *post hoc* Tukey test. Figure S2: A10, a specific antagonist for TRPM2, accelerates autophagic flux under OGD-R treatment. (a) SH-SY5Y cells overexpressing mRFP-GFP-LC3 were treated with OGD for 6 h followed by 6 h of reperfusion. 10 *μ*M A10 was added during reperfusion. (b) The mean number of red LC3 puncta per cell in each condition was quantified. All data are from 3 independent experiments. Error bars: SEM. ^∗∗^*p* < 0.01, ^∗∗∗∗^*p* < 0.0001; one-way ANOVA with post hoc Tukey test (Supplementary Material).
