# Supplementary material for: Deficiency of ROS-Activated TRPM2 Channel Protects Neurons from Cerebral Ischemia-Reperfusion Injury through Upregulating Autophagy
Source: Oxid Med Cell Longev. 2021 Jul 27;2021:7356266. doi: 10.1155/2021/7356266 (PMC8337124; doi:10.1155/2021/7356266)
Supplement: Supplementary Materials — Figure S1:TRPM2 deficiency-upregulated autophagy is Beclin1-independent. (a) Primary cultured neurons subjected to medium alone (CTRL), 1 hour of OGD plus 3 hours of reperfusion (OGD-R) at DIV9. 3 μg of protein was sequentially immunoblotted with antibodies against Beclin1, phosphorylated-S295-Beclin1, and β-actin. (b) Normalized ratio of the phosphorylated-S295-Beclin1 and Beclin1 band intensities, determined from 3 independent experiments performed as in (a). ∗∗∗∗p < 0.0001; NS: not significant; one-way ANOVA with post hoc Tukey test. Figure S2: A10, a specific antagonist for TRPM2, accelerates autophagic flux under OGD-R treatment. (a) SH-SY5Y cells overexpressing mRFP-GFP-LC3 were treated with OGD for 6 h followed by 6 h of reperfusion. 10 μM A10 was added during reperfusion. (b) The mean number of red LC3 puncta per cell in each condition was quantified. All data are from 3 independent experiments. Error bars: SEM. ∗∗p < 0.01, ∗∗∗∗p < 0.0001; one-way ANOVA with post hoc Tukey test (Supplementary Material). [file 7356266.f1.pdf]

Supplementary Materials

Oxidative Medicine and Cellular Longevity

Special Issue on

Reactive Oxygen Species in inflammation and Tissue Injury

Deficiency of ROS-activated TRPM2 ion channel protects neurons from cerebral ischemia-reperfusion injury through up-regulating autophagy

Xupang Hu[1,#], Lijuan Wu[1,2,#], Xingyu Liu[1], Yi Zhang[1], Min Xu[1], Qiuyuan Fang[1], Lin Lu[3], Tarek Mohamed Abd El-Aziz[4,5], Fangfang Li[1,*], Wei Yang[1,*]

[1]Department of Biophysics, and Department of Neurosurgery of the First Affiliated Hospital, Zhejiang University School of Medicine, Hangzhou 310058, China;

[2]School of Medicine, Taizhou University, Taizhou, China

[3]School of Medicine, Zhejiang University City College, Hangzhou 310015, China;

[4]Department of Cellular and Integrative Physiology, University of Texas Health Science Center at San Antonio, San Antonio, TX 78229-3900, USA;

[5]Zoology Department, Faculty of Science, Minia University, El-Minia 61519, Egypt

These authors contributed equally to this work.

Correspondence should be addressed to: Wei Yang; yangwei@zju.edu.cn

Fangfang Li; 0618314@zju.edu.cn

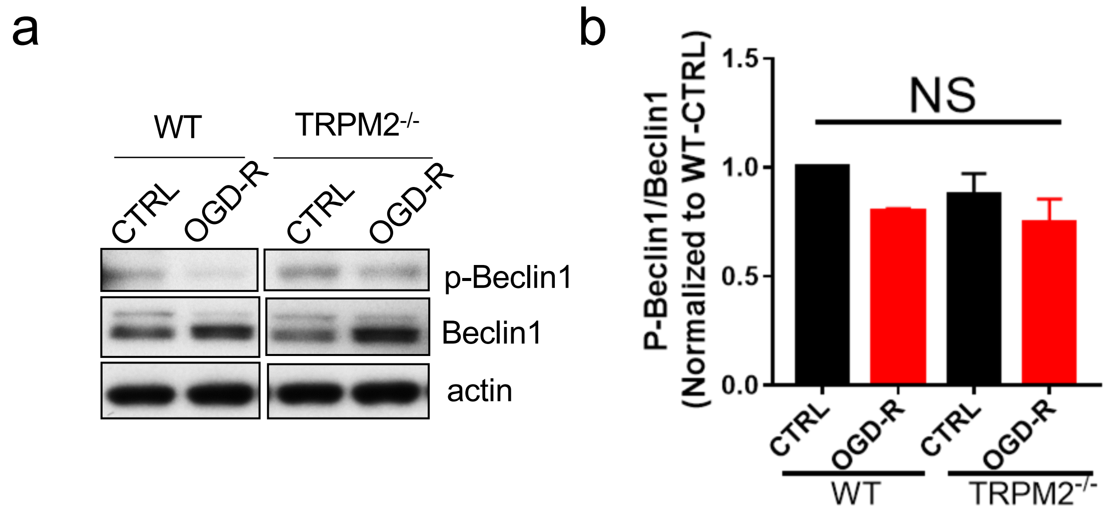

Figure S1. TRPM2 deficiency-upregulated autophagy is Beclin1-independent. (a) Primary cultured neurons subjected to medium alone (CTRL), 1 h of OGD plus 3 h of reperfusion (OGD-R) at DIV9. 3 μg of protein was sequentially immunoblotted with antibodies against Beclin1, phos-S295-Beclin1 and β-actin. (b) Normalized ratio of the phos-S295-Beclin1 and Beclin1 band intensities, determined from three independent experiments performed as in (a). **** $p<0.0001$; NS, not significant; One-way ANOVA with *post-hoc* Tukey test.

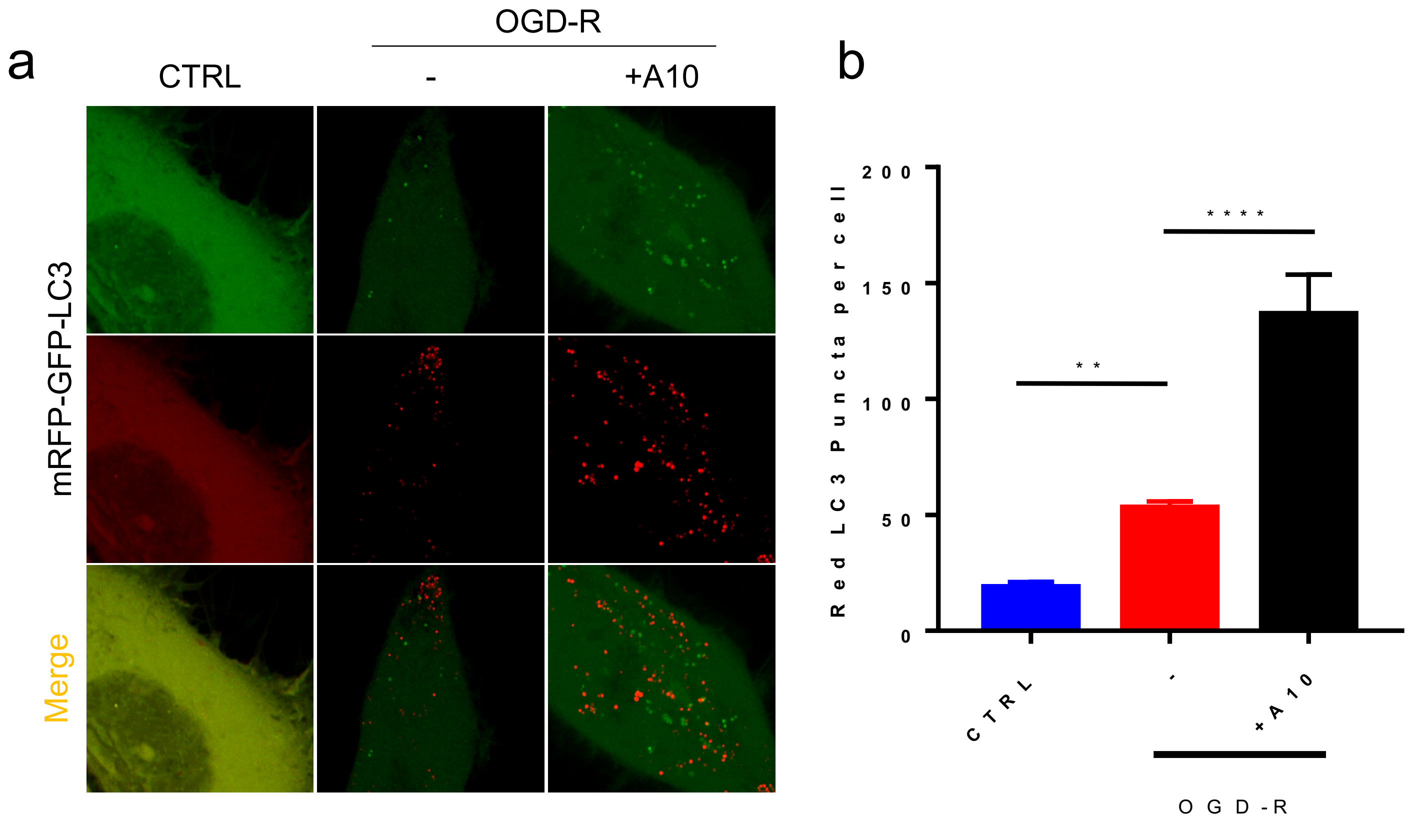

Figure S2. A10, a specific antagonist for TRPM2, accelerates autophagic flux under OGD-R treatment.(a) SH-SY5Y cells overexpressing mRFP-GFP-LC3 were treated with OGD for 6 h followed by 6 h of reperfusion. 30 μ M A10 was added during reperfusion. (b) The mean number of red LC3 puncta per cell in each condition were quantified. All data are from 3 independent experiments. Error bars: SEM. One-way ANOVA with post-hoc Tukey test. ** indicates p<0.01, **** indicates p<0.0001.
